# Factors Associated with Recent HIV Testing among Heterosexuals at High Risk for HIV Infection in New York City

**DOI:** 10.3389/fpubh.2016.00076

**Published:** 2016-04-27

**Authors:** Marya Gwadz, Charles M. Cleland, Alexandra Kutnick, Noelle R. Leonard, Amanda S. Ritchie, Laura Lynch, Angela Banfield, Talaya McCright-Gill, Montserrat del Olmo, Belkis Martinez

**Affiliations:** ^1^Center for Drug Use and HIV Research (CDUHR), New York University College of Nursing, New York, NY, USA

**Keywords:** HIV testing, HIV screening, heterosexuals, barriers to HIV testing, structural barriers, health disparities, sex differences

## Abstract

**Background:**

The Centers for Disease Control and Prevention recommends persons at high risk for HIV infection in the United States receive annual HIV testing to foster early HIV diagnosis and timely linkage to health care. Heterosexuals make up a significant proportion of incident HIV infections (>25%) but test for HIV less frequently than those in other risk categories. Yet factors that promote or impede annual HIV testing among heterosexuals are poorly understood. The present study examines individual/attitudinal-, social-, and structural-level factors associated with past-year HIV testing among heterosexuals at high risk for HIV.

**Methods:**

Participants were African-American/Black and Hispanic heterosexual adults (*N* = 2307) residing in an urban area with both high poverty and HIV prevalence rates. Participants were recruited by respondent-driven sampling in 2012–2015 and completed a computerized structured assessment battery covering background factors, multi-level putative facilitators of HIV testing, and HIV testing history. Separate logistic regression analysis for males and females identified factors associated with past-year HIV testing.

**Results:**

Participants were mostly male (58%), African-American/Black (75%), and 39 years old on average (SD = 12.06 years). Lifetime homelessness (54%) and incarceration (62%) were common. Half reported past-year HIV testing (50%) and 37% engaged in regular, annual HIV testing. Facilitators of HIV testing common to both genders included sexually transmitted infection (STI) testing or STI diagnosis, peer norms supporting HIV testing, and HIV testing access. Among women, access to general medical care and extreme poverty further predicted HIV testing, while recent drug use reduced the odds of past-year HIV testing. Among men, past-year HIV testing was also associated with lifetime incarceration and substance use treatment.

**Conclusion:**

The present study identified gaps in rates of HIV testing among heterosexuals at high risk for HIV, and both common and gender-specific facilitators of HIV testing. Findings suggest a number of avenues for increasing HIV testing rates, including increasing the number and types of settings offering high-quality HIV testing; promoting STI as well as HIV testing; better integrating STI and HIV testing systems; implementing peer-driven social/behavioral intervention approaches to harness the positive influence of social networks and reduce unfavorable shared peer norms; and specialized approaches for women who use drugs.

## Introduction

Since 2006, the Centers for Disease Control and Prevention (CDC) has recommended persons at high risk for HIV infection in the United States (US) receive diagnostic HIV testing annually, to foster early HIV diagnosis, timely linkage to HIV care, and modification of behaviors that potentially place others at risk for contracting HIV (1). In response, rates of lifetime HIV testing in the US have increased modestly, although fewer than half in the general population (44%) has ever been tested for HIV (2). African-Americans/Blacks have the highest rates of lifetime HIV testing (63.9%), followed by Non-Hispanic Whites (42.9%), then Mexican Americans (35.7%) (2). Further, those with a non-heterosexual identity (that is, lesbian, gay, bisexual, or other non-heterosexual identity) have higher lifetime HIV testing rates than heterosexuals (57.3 vs. 44.2%) (2). Yet heterosexuals make up a significant proportion of new HIV infections in the US (27%) (3) and the promotion of HIV testing among heterosexuals, particularly those at high risk for HIV infection, is a public health priority (1).

The factors that promote or impede HIV testing among high-risk heterosexuals are under-studied compared to other risk groups such as men who have sex with men (MSM) and persons who inject drugs (4, 5). A primary barrier to the study of heterosexuals at high risk (HHR) has been the lack of an accepted definition of the population (6, 7). The CDC National HIV Behavioral Surveillance (NHBS) system defines HHR for HIV as persons linked within urban geographical areas with elevated rates of heterosexually transmitted HIV, which also have very high rates of poverty (8). Further, African-American/Black and Hispanic populations are greatly over-represented in these “high-risk areas” compared to Whites (9). Yet rates of annual HIV testing are low among HHR: in New York City, only 31% of men and 35% of women had tested in the past year, although more than 90% had encountered settings where HIV testing was offered. This pattern suggests HHR frequently decline HIV testing, and, therefore, that access to high-quality services that motivate HHR to engage in HIV testing are lacking (10). As a result of these low HIV test rates, late diagnosis of HIV infection is common in this group (11–13). In response, the CDC has called for research to identify barriers to HIV testing for HHR, in order to inform future policies, culturally appropriate interventions, and HIV testing initiatives (14).

The present study explores a set of putative barriers to/facilitators of past-year HIV testing among African-American/Black and Hispanic HHR in New York City. Grounded in the literature which highlights the multiple factors that explain HIV testing in high-risk populations (15, 16), we conceptualize these potential factors within the Theory of Triadic Influence (17). The Theory of Triadic Influence is an integrative social-cognitive theory emphasizing three “streams of influence” on health behavior, namely at the individual/attitudinal-, social-, and structural levels, the latter receiving relatively little attention to date in the literature on HIV testing (18). Within the framework of this theory, the primary specific factors believed to influence the HIV testing behavior of HHR are summarized briefly below and described in more detail elsewhere (19).

Individual/attitudinal barriers to annual HIV testing include lack of awareness of recommended HIV testing frequency, low perceived risk of HIV (20), fear of HIV and its consequences including stigma (21, 22), and distrust of medical environments (21). Further, substance use serves as a barrier to HIV testing (21, 23), as do other “competing priorities,” such as mental health problems and unstable housing, complicated by low socioeconomic status (20, 24). At the social level of influence, insufficient social support (25) and perceived peer norms that do not support HIV testing can impede HIV testing (26). At the structural level of influence, HHR often have inadequate access to settings where high-quality HIV testing is offered (10, 13). Theoretically, barriers at these three levels of influence combine and interact to impede access to and motivation for HIV testing among HHR. At the same time, HHR evidence factors facilitating HIV testing, such as intrinsic motivation to achieve good health, and involvement in health-care and other settings that provide services (10, 27). Yet men and women differ in their need for and/or access to health-care settings, for example, related to higher rates of incarceration among men, and receipt of gynecological and prenatal care for many women (10), suggesting the need to explore gender differences. The present study seeks to advance the literature on factors impeding or promoting past-year HIV testing in a population at-risk by examining barriers at multiple levels of influence, including structural-level factors, and exploring gender differences.

## Materials and Methods

### Design

Participants were recruited in 2012–2015 using respondent-driven sampling (RDS) (28, 29), a peer-to-peer social network-based recruitment method, as part of a larger study to test intervention approaches to uncover undiagnosed HIV infection among HHR in New York City. The study was approved by the Institutional Review Board of the New York University Langone School of Medicine.

### Study Setting

**Figure 1:**
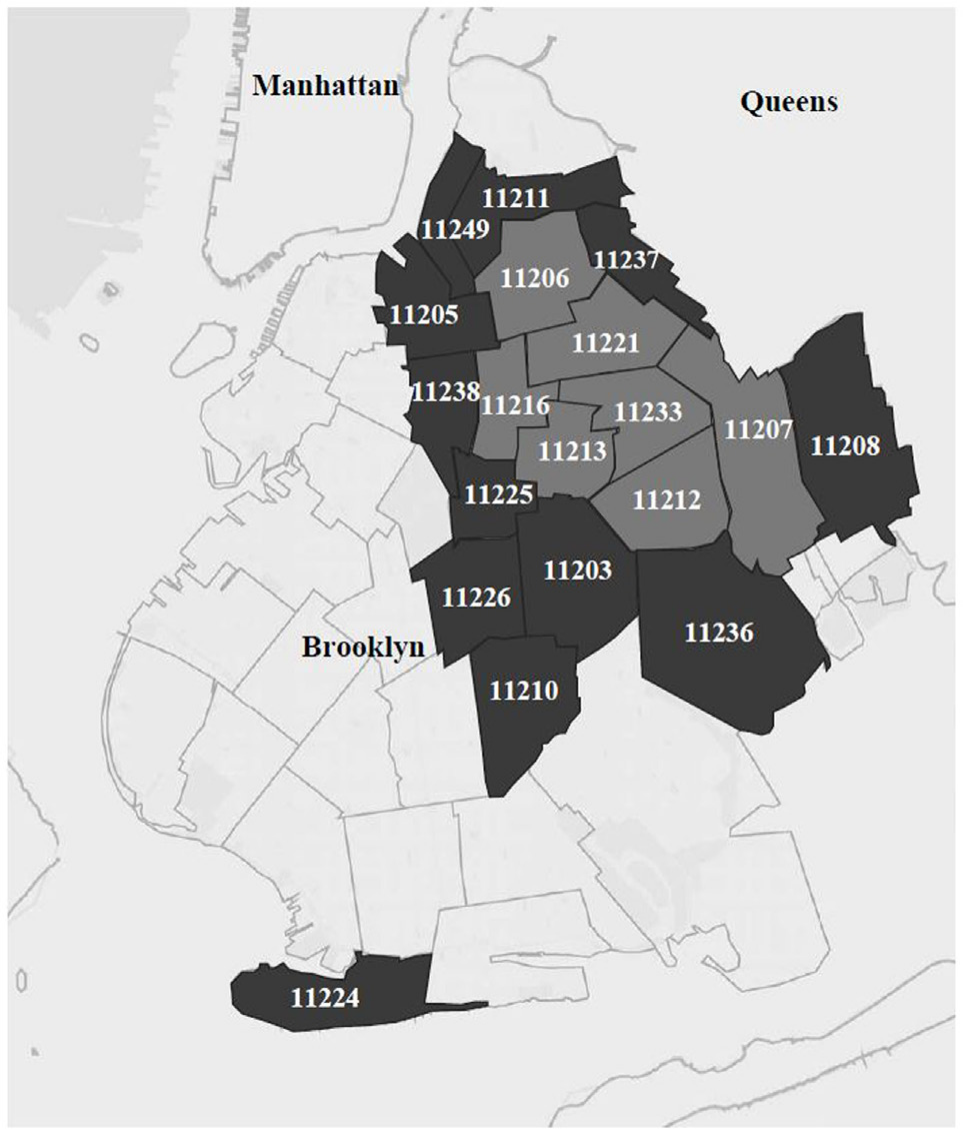
**Core high-risk area (“HRA,” in light gray) and surrounding larger HRA, in the borough of Brooklyn in New York City**.

Study procedures were modeled on the NHBS system with HHR (8) and were described in detail elsewhere (19). The study was located in a high-risk area (HRA) defined within Brooklyn, the borough in New York City with the highest heterosexual HIV prevalence at the time the study was planned (19). The HRA for the present study comprised two regions: a core area made up of the top 25% of zip codes ranked by heterosexual HIV prevalence and household poverty (seven zip codes, see the light gray area in Figure 1). Further, because the social networks of HHR cross zip code boundaries, the HRA also included the next quartile of zip codes (dark gray area in Figure 1). This was intended to maintain the study’s focus on this core HRA while reducing artificial restriction of RDS recruitment chains. The study field site was located in the core HRA, as was recruitment of the initial participants who started the RDS recruitment chains. However, recruitment of peers during RDS could extend to the larger HRA.

### Sampling and Recruitment

RDS is designed to reach deep into hidden or wary populations, engaging more isolated network members who may not be present or easy to engage in typical social venues (30). RDS begins with recruitment of “initial seeds,” who then recruit a small number of their peers for the study. Next, peer-to-peer recruitment continues until sample size goals are met. Each set of peers recruited is called a “recruitment wave.” In the present study, a total of 107 initial seeds, selected to vary in age, gender, race/ethnicity, and substance use history, were directly recruited by staff in 2012–2014 from public and street venues within a core HRA, and enrolled into the study. Each seed could start a recruitment chain by recruiting three to five of their peers (47% of initial seeds recruited ≥1 peer), and these peers then entered the study and recruited their own peers until the target sample size was achieved. The average number of waves on these recruitment chains was 7 (range 1–26 waves/chain). Overall 66% of participants (including both initial seeds and peers) recruited at least one individual. Both seeds and peers are included as participants in the present study, but some participants were excluded if they were screened for the study prior to a change made in inclusion criteria related to HIV testing history (*n* = 383) described in more detail elsewhere (19) or were missing data on variables needed for analysis (*n* = 304). Thus, the sample size for the present study was 2307 participants.

### Eligibility

Study eligibility criteria for the initial seeds were 18–60 years; sexually active (vaginal and/or anal sex) with at least one opposite sex partner within the previous year; reside in the core HRA in central Brooklyn; African-American/Black or Hispanic race/ethnicity (to focus on the populations with the greatest barriers to regular HIV testing); comprehension of English or Spanish; unknown or negative HIV status; and not actively psychotic based on a standard screening instrument (31, 32). Eligibility criteria for peers were the same with two exceptions: peers could reside in the core or larger HRA (to reduce artificial restriction on recruitment chains), and peers could have an HIV-negative, unknown, or HIV-positive status (because HIV-infected individuals can be a conduit to those with undiagnosed HIV infection, but including HIV-infected individuals as initial seeds could potentially lead to an over-presentation of HIV-infected participants in the study). The present paper excludes those enrolled who were previously diagnosed with HIV (*N* = 116).

### Procedures and Measures

#### Enrollment and Assessment

With the exception of initial seeds, participants were recruited by peers and presented to the study field site with a coded recruitment coupon linking them back to the recruiter. Potential participants, both initial seeds and peers, provided verbal consent and were screened for eligibility with a brief structured assessment (10 min). Those found eligible provided signed informed consent for remaining study activities and then completed a structured baseline interview. The interview lasted 60–90 min and was administered by trained staff on computers using an audio, computer-assisted self-interviewing (ACASI) program (33). Participants received compensation of $15 for the screening and $30 for the baseline interview, as well as funds for round-trip local transportation. The recruiter received $15 for each peer referred and found eligible.

#### Measures

The measures used in the present study were drawn primarily from a set of harmonized instruments used for the set of “Seek, Test, Treat, and Retain” studies sponsored by the National Institute on Drug Abuse (NIDA) at the National Institutes of Health (34). These measures have been used in past studies with HHR and similar vulnerable populations and are described in brief below (Cronbach’s alpha provided for scales where appropriate).

##### Sociodemographic and Background Factors and Barriers and Facilitators

###### Sociodemographic and Background Factors

Using a structured NIDA-harmonized instrument (35), we assessed age (in years), race/ethnicity (African-American/Black and Latino/Hispanic), and gender (coded as male gender, yes/no). The remaining sociodemographic indices were coded to reflect the predominant categories and were coded as yes/no, with the “yes” value presented in the tables below for parsimony. These included marital status (married or living as married or in a long-term relationship), whether has children and whether children <10 years old live at home, heterosexual identity (because some participants met eligibility criteria and identified as bisexual), education level, namely, whether the participant has achieved a high-school (HS) diploma or completed HS or a General Educational Development (GED) test but no college, employed full or part-time, financial insecurity (i.e., unable to pay for necessities in the past year), any health insurance, housing status (that is, whether has ever been homeless and is currently homeless), and history of incarceration (ever incarcerated and if so, whether incarcerated in the past year).

###### Sexual Behavior and Involvement in Sex Work

The NHBS measure (36) was used to gather data on lifetime and past month sexual behavior and history, coded as yes/no or presented as a mean and standard deviation SD. These included whether participants had a same sex partner over their lives (yes/no), engaged in a group sex event over the lifetime (yes/no), the number of sexual partners past month (mean, SD), whether had sex without a condom past month (yes/no), and whether, over the lifetime, engaged in transactional sex such as giving money/drugs to and/or receiving money/drugs from a partner in exchange for sex.

###### Drug and Alcohol Use

Using NIDA-harmonized instruments (37), we assessed drug use in the past year (yes/no) and past month (yes/no) across 12 different substances, the frequency of drug use in the past month on an 8-point Likert-type scale ranging from never through 10 or more times a day/almost every day (mean, SD) (38). Using recognized screening measures (TCU Drug Screen and AUDIT) and established cut-off values for problematic levels of use, we assessed the presence or absence of drug use problems in the past year [nine items; Cronbach’s alpha (α) = 0.91] (39); as well as alcohol problems in the past year (10 items; α = 0.89) (40), coded as problematic drug or alcohol use (yes/no), and the proportion who experienced either a drug or alcohol problem in the past year. Substance use treatment over the lifetime, an indication of substance use problems, was assessed (e.g., detoxification, rehabilitation, outpatient treatment, and 12-step programs) and coded as present or absent across all categories. Daily cigarette smoking was assessed, as was lifetime and past-month history of injection drug use (37).

###### Physical and Mental Health

We assessed general health using the RAND-36 (five items, α = 0.77; range 0–100, with higher scores indicating better perceived health) (41); a history of sexually transmitted infection (STI) testing (other than HIV testing) and STI diagnosis over the lifetime (38); depressive symptoms at a clinically significant level over the past week (20-item CES-D; α = 0.80) (42); and current general anxiety at a clinically significant level (six-item BSI anxiety; α = 0.88) (43). Composite depression and anxiety scores were calculated and cut-offs of 16 or greater (depression) and 0.7 or greater (anxiety) were used to determine presence or absence of symptoms at a clinically significant level.

###### Individual/Attitudinal-Level Factors

HIV knowledge was assessed with the HIV-KQ-18 (18 true/false items; α = 0.81, e.g., “People who have been infected with HIV quickly show serious signs of being infected”) (44); distrust of health care (seven items; assessed on a 5-point Likert-type scale; α = 0.72, e.g., “Sometimes I wonder if healthcare organizations really know what they are doing”) (45); and HIV fears and conspiracy beliefs (five items; α = 0.69, assessed on a 5-point Likert-type scale; e.g., “There is a cure for HIV or AIDS but the government is keeping it from us”) (46). For each scale, after re-coding reverse-coded items as appropriate, items were summed (knowledge) or averaged (distrust and fears/conspiracy beliefs) to provide scale scores, and a mean and SD calculated. We assessed health literacy (one item, “How confident are you filling out medical forms by yourself?” where participants with less confidence were contrasted with extremely confident participants) (47).

##### Social-Level Factors

We assessed social support (five items; assessed on a 5-point Likert-type scale; α = 0.88, e.g., “extent to which you have someone to love and make you feel wanted?”) (41); and perceived peer norms in support of HIV testing (seven items; assessed on a 7-point Likert-type scale and reverse-coded as appropriate; α = 0.64; including descriptive norms, that is, an individual’s perceptions of what other people are doing; e.g., “How many of your close friends and family have ever had an HIV test?,” and injunctive or proscriptive norms, that is, perceptions of what behaviors other people consider acceptable; e.g., “How many of your close friends and family have encouraged you to get an HIV test?”) (48). For both of these scales, after re-coding reverse-coded items as appropriate, items were averaged.

##### Structural-Level Factors

We assessed ability to access general medical care when needed as a single item (yes/no) (49); overall barriers to general medical care (10 items; coded yes/no; α = 0.79, e.g., not sure where to go to get medical care, did not have transportation to medical care; summed with a range of 0–10) (50); and perceived ease of access to HIV testing, a scale that included organizational factors, such as ease of access, convenience, professionalism, and confidentiality, as well as emotional/attitudinal barriers that impede access, such as fatalism and fear and mistrust of HIV testing (14 items; assessed on a 5-point Likert-type scale α = 0.81; averaged, resulting in a range of 0–4) (50).

#### Data Analysis

Logistic regression was used to estimate bivariate associations between predictors and HIV testing in the past year separately for women and men. Logistic regression was also used to estimate associations in multivariable models separately for women and men. Multivariable models started with main effects of 47 potential predictors of HIV testing in the past year (all variables in Table 1 except for gender and recent HIV testing). Starting with terms furthest from significance, terms were removed if the associated *p*-value (*p*) was greater than 0.10. This backward elimination of non-significant terms used the method of Lawless and Singhal (51) implemented in the *rms* package (52) of the R statistical computing environment (53), which was used for all analyses.

**Table 1:** **Participant characteristics**.

	% or mean (SD)
**Sociodemographic and background factors**	
HIV test in the past year	50.41
Male gender	57.56
Age (years)	39.10 (12.06)
African-American/Black	73.95
Latino/Hispanic	23.93
Married, living as married	21.72
In a long-term relationship	34.72
Has any children	67.71
Children <10 living at home	20.03
Identifies as heterosexual	88.21
No high school diploma	35.98
Completed HS or GED but no college	41.09
Employed full or part-time	19.38
Ran out of money for basic necessities past 12 months	80.84
Any health insurance	85.52
Ever homeless	54.49
Currently homeless	21.37
Ever incarcerated	61.94
Past year incarceration if ever incarcerated	23.02
**Sexual behavior and involvement in sex work**	
Lifetime same sex partner(s)	18.73
Lifetime group sex	18.16
Number of sex partners past month	1.72 (3.92)
Sex without a condom past month	59.56
Lifetime gave money/drugs for sex	23.49
Lifetime received money/drugs for sex	31.04
**Drug and alcohol use and problems**	
Any drug use in the past year	46.03
Any drug use in the past month	32.77
Drug use frequency past month (0–8)	1.34 (2.38)
Meets TCU criterion for drug problem past year	21.15
Meets AUDIT criterion for alcohol problem past year	25.88
Meets criterion for drug or alcohol problem past year	36.93
Ever in substance use treatment	42.78
Daily cigarette smoking in the past month	53.97
Ever injected drugs not for a medical reason	9.06
Injected drugs in the past 30 days	1.30
**Physical and mental health**	
RAND-36 general health	70.12 (19.75)
STI testing lifetime	66.67
STI diagnosis lifetime	25.88
CESD-20 depression total score ≥16	57.48
BSI anxiety ≥0.70	57.65
**Individual/attitudinal-level factors**	
HIV knowledge (range 0–18)	12.67 (3.66)
Mistrust of health care (range 0–4)	2.02 (0.69)
HIV conspiracy beliefs (range 0–4)	1.47 (0.46)
Low health literacy	51.97
**Social-level factors**	
Social support (range 0–4)	2.21 (1.14)
Peer norms in support of HIV testing (range 0–6)	4.55 (0.95)
**Structural-level factors**	
Able to get medical care whenever needed	49.59
Barriers to care total (range 0–10)	1.51 (2.07)
HIV testing access (range 0–4)	3.41 (0.56)

## Results

### Participant Characteristics

Table 1 shows sociodemographic and background factors, sexual behavior, drug and alcohol use and problems, physical and mental health, individual-attitudinal factors, social-level factors, and structural-level factors. About 58% of participants were male, and most (75%) were non-Hispanic African-Americans/Blacks. Age ranged from 18 to 60 years, with a mean age of 39.10 years (SD = 12.06 years). Many (36%) had not completed HS or attained a GED, and more than half (54%) had experienced homelessness. More than half (62%) had been incarcerated, 23% within the past year. More than half (60%) had sex without a condom in the past month, and more than a quarter (28%) had more than one sex partner in the past month. Receiving money or drugs for sex was not uncommon (30%). About one in five participants (21%) met criteria for a current drug problem, and 37% met criteria for a current alcohol or drug problem. Many (43%) had received substance use treatment in the past. Over half (57%) had depressive symptoms at a clinically significant level, as well as anxiety (58%), and most (72%) met criteria for either a current anxiety or depression problem (not in Table 1). Although most (86%) had health insurance, half (50%) reported they could not get medical care whenever they needed it.

Half reported testing for HIV within the past year (50%), although most had tested for HIV at least once prior to the study (93%). To assess regularity of HIV testing since CDC’s annual HIV testing recommendation, we calculated the ratio of number of lifetime HIV tests to number of years in which the participant was at least 18 years of age. This ratio ranged from 0 to 56, with a median of 0.74. Thus, most participants (63%) reported fewer HIV tests than the number of years since the annual HIV testing recommendation began, indicating only 37% engaged in regular, annual HIV testing.

### Predictors of HIV Testing in the Past Year

Table 2 shows bivariate associations between testing for HIV in the past year and other variables, by gender. Among women, older age [odds ratio (OR) = 0.82], drug use in the past month (OR = 0.74), drug use frequency in the past month (OR = 0.85), having elevated anxiety (OR = 0.73), HIV conspiracy beliefs (OR = 0.82), low health literacy (OR = 0.59), and barriers to care (OR = 0.84) were each negatively associated with HIV testing in the past year (*p* < 0.05). Also among women, young children at home (OR = 1.35), health insurance (OR = 1.99), lifetime homelessness (OR = 1.44), better general health (OR = 1.15), lifetime STI testing (OR = 2.56), more knowledge of HIV (OR = 1.40), social support (OR = 1.24), peer norms in support of HIV testing (OR = 1.43), better access to medical care (OR = 1.87), and better access to HIV testing (OR = 1.75) were positively associated with HIV testing in the past year (*p* < 0.05).

**Table 2:** **Factors associated with recent HIV testing among female and male heterosexuals at high risk in New York City (*n* = 2307)**.

	Female (***n*** = 979)			Male (***n*** = 1328)		
	No recent HIV test (***n*** = 440)	HIV test in past 12 months (***n*** = 539)	Female bivariate odds ratio	No recent HIV test (***n*** = 704)	HIV test in past 12 months (***n*** = 624)	Male bivariate odds ratio
		
	Mean (SD) or %	Mean (SD) or %		Mean (SD) or %	Mean (SD) or %	
**Sociodemographic and background factors**						
Age^a^	39.65 (12.13)	36.42 (11.81)	0.82**	40.07 (12.41)	39.94 (11.52)	1.03
Children <10 living at home	27.05	36.36	1.35*	11.79	10.26	0.80
Any health insurance	87.27	93.14	1.99**	79.40	84.62	1.41*
Ever homeless	49.32	56.22	1.44**	51.28	60.26	1.44**
Currently homeless	17.05	19.67	1.25	20.45	26.92	1.50**
Past year incarceration	11.82	13.36	1.42	27.13	34.62	1.57***
**Sexual behavior and involvement in sex work**						
Lifetime same sex partner(s)	36.14	32.10	0.83	7.53	7.53	1.05
Lifetime group sex	15.68	15.58	0.98	18.89	21.31	1.10
Number of sex partners past month^a^	1.66 (3.56)	1.29 (2.04)	0.86	2.06 (4.04)	1.74 (2.96)	0.92
Sex without a condom past month	61.36	64.56	1.07	55.97	58.01	0.98
Lifetime gave money/drugs for sex	8.41	4.64	0.59	36.93	35.26	0.95
Lifetime received money/drugs for sex	27.73	26.90	1.07	31.82	36.06	1.25
**Drug and alcohol use and problems**						
Any drug use in the past month	31.14	23.93	0.74*	39.49	33.97	0.79
Drug use frequency past month (0–8)^a^	1.23 (2.32)	0.89 (2.01)	0.85*	1.68 (2.57)	1.42 (2.42)	0.91
Meets criterion for drug or alcohol problem	35.00	32.47	0.99	36.93	42.15	1.31*
Ever in substance use treatment	31.59	31.73	1.09	47.16	55.29	1.51***
Daily cigarette smoking in the past month	49.09	50.83	1.11	54.12	59.94	1.28*
Ever injected drugs not for a medical reason	7.05	5.01	0.75	10.94	11.86	1.14
**Physical and mental health (general, sti)**						
RAND-36 general health^a^	68.03 (20.44)	71.55 (19.81)	1.15*	69.36 (19.63)	71.20 (19.20)	1.07
STI testing lifetime	69.32	85.34	2.56***	49.86	67.63	2.16***
CESD-20 depression total score ≥16	61.36	57.33	0.92	55.97	56.57	1.01
BSI anxiety ≥0.70	60.23	50.65	0.73*	59.94	59.29	0.95
**Individual/attitudinal-level factors**						
HIV knowledge (0–18)^a^	12.23 (3.68)	13.48 (3.12)	1.40***	12.02 (3.96)	13.00 (3.55)	1.27***
Mistrust of health care (0–4)^a^	2.04 (0.69)	1.98 (0.67)	0.93	2.02 (0.72)	2.05 (0.66)	1.07
HIV conspiracy beliefs (0–4)^a^	1.43 (0.76)	1.30 (0.69)	0.82**	1.56 (0.80)	1.53 (0.75)	0.99
Low health literacy	52.27	37.11	0.59***	61.51	53.85	0.74*
**Social-level factors**						
Social support (0–4)^a^	2.19 (1.14)	2.49 (1.12)	1.24**	2.09 (1.14)	2.12 (1.12)	1.05
Peer norms about HIV testing (0–6)^a^	4.46 (1.01)	4.82 (0.86)	1.43***	4.32 (0.99)	4.64 (0.88)	1.42***
**Structural-level factors**						
Able to get medical care whenever needed	49.77	65.49	1.87***	39.63	46.96	1.40**
Barriers to care total (0–10)^a^	1.64 (2.18)	0.95 (1.42)	0.84***	1.73 (2.25)	1.64 (2.17)	0.99
HIV testing access (0–4)^a^	3.32 (0.58)	3.60 (0.47)	1.75***	3.23 (0.60)	3.50 (0.49)	1.65***

*^a^Odds ratios for these variables reflect the expected change in odds of recent testing for a 1 SD increase in the variable*.

*p < 0.05.

**p < 0.01.

***p < 0.001.

Among men, low health literacy (OR = 0.74) was negatively associated with HIV testing in the past year (*p* < 0.05). Also among men, health insurance (OR = 1.41), lifetime or past year homelessness (OR = 1.44), past-year incarceration (OR = 1.57), meeting criteria for a drug or alcohol problem (OR = 1.31), lifetime substance use treatment (OR = 1.51), daily cigarette smoking (OR = 1.28), lifetime STI testing (OR = 2.16), and more knowledge of HIV (OR = 1.27) were each positively associated with HIV testing in the past year (*p* < 0.05).

Table 3 shows adjusted associations between testing for HIV in the past year and other variables. Among women, lifetime STI testing [adjusted odds ratio (AOR) = 1.99] and diagnosis (AOR = 2.21), peer norms in support of HIV testing (AOR = 1.22), better access to medical care (AOR = 1.57), better access to HIV testing (AOR = 1.48), and running out of money for basic necessities in the past year (AOR = 1.53) were each positively associated with testing for HIV in the past year. An alcohol or drug problem also was positively associated with testing for HIV in the past year (AOR = 1.35) but with only marginal statistical significance. Drug use frequency in the past 30 days was negatively associated with testing for HIV in the past year (AOR = 0.81). Among men, lifetime STI testing (AOR = 1.46) and diagnosis (AOR = 2.08), peer norms in support of HIV testing (AOR = 1.23), better access to HIV testing (AOR = 1.52), and lifetime substance use treatment (AOR = 1.66) were each positively associated with testing for HIV in the past year. Also, both men who were never incarcerated (AOR = 1.60) and men who were incarcerated within the past year (AOR = 1.74) were more likely to have tested for HIV in the past year when compared to men who reported lifetime incarceration but had not been incarcerated in the past year.

**Table 3:** **Factors associated with recent HIV testing among female and male heterosexuals at high risk in New York City: multivariate logistic regression**.

	Female			Male		
	AOR	95% CI	*p*-value	AOR	95% CI	*p*-value
Sexually transmitted infections						
Tested, never diagnosed, vs. never tested	1.99	(1.34–2.98)	0.0008	1.46	(1.06–2.02)	0.0199
Tested, diagnosed, vs. never tested	2.21	(1.53–3.21)	<0.0001	2.08	(1.57–2.76)	<0.0001
HIV testing peer norms^a^	1.22	(1.05–1.43)	0.0114	1.23	(1.08–1.41)	0.0018
HIV testing access^a^	1.48	(1.25–1.77)	<0.0001	1.52	(1.33–1.75)	<0.0001
Able to get medical care when needed	1.57	(1.16–2.11)	0.0030			
Ran out of money for basic necessities past 12 months	1.53	(1.09–2.16)	0.0151			
Drug use frequency past 30 days	0.81	(0.69–0.95)	0.0116			
Alcohol or drug problem	1.35	(0.98–1.87)	0.0703			
Incarceration						
Never vs. lifetime but not past 12 months				1.60	(1.16–2.22)	0.0040
Past 12 months vs. lifetime but not past 12 months				1.74	(1.31–2.31)	0.0002
Ever in substance use treatment				1.66	(1.28–2.15)	0.0001

*^a^Adjusted odds ratios for these variables reflect the expected change in odds of recent testing for a 1 SD increase in the variable*.

Because lifetime STI testing had the strongest unique association with recent HIV testing in the multivariable models for both female and male participants, and because STI testing may be related to a number of the other variables considered as potential determinants of HIV testing, we excluded lifetime STI testing as a sensitivity analysis for the results presented in Table 3. For females, excluding lifetime STI testing allowed HIV knowledge to enter the final multivariable model. HIV knowledge was positively associated with recent HIV testing (AOR = 1.24, 95% CI: 1.06–1.46, *p* = 0.0081). For males, excluding lifetime STI testing did not lead to any other changes to the variables included in the final multivariable model presented in Table 3.

## Discussion

This study advances what is known about HIV testing patterns among an under-studied population of adults at high risk for HIV in the US – heterosexuals who reside in urban HRAs with elevated poverty and HIV prevalence rates. Participants in this sample evidenced numerous serious risk factors for HIV infection and other adverse psychosocial outcomes, such as high rates of unemployment, homelessness, incarceration, and substantial rates of substance use problems, as well as protective factors. For example, almost all had health insurance and half were married or in long-term stable relationships, suggesting the availability of social support. We found half of those enrolled had been tested for HIV in the past year, and 37% evidenced regular, annual HIV testing. While these HIV testing rates do not yet meet the CDC’s goals of universal or near universal coverage, they are substantially higher than a recent local NHBS study with HHR that used the same sampling method and an equivalent definition of the population, where approximately a third had received past-year HIV testing (10). In light of national and local policies and HIV testing initiatives put in place to increase HIV testing among those at risk, including the routinizing HIV testing, study findings may therefore reflect upward trends in annual HIV testing rates among HHR (54, 55). (Alternately, they may reflect methodological differences between the present study and NHBS.) Yet, despite these gains, most HHR do not test annually, and they are substantially less likely to be tested annually than their MSM peers. In fact, a recent NHBS study of MSM recruited in New York City found about three quarters (76%) had tested within the past year (4), highlighting the importance of understanding factors that promote or impede HIV testing among HHR.

### Sociodemographic, Background, and Health-Related Factors

Among women, the frequency of drug use reduced the odds of past-year HIV testing. Drug use often results in “competing priorities,” which thereby reduce engagement in health-care behavior and also creates experiences of stigma for the drug user (56). Stigma operates by raising the specter of serious potential consequences associated with the socially undesirable behavior or condition, including rejection by friends and family, loss of a job or housing, discrimination, and violence (57). For female HHR who use drugs, who may also be represented in other stigmatized categories (e.g., poor, formerly incarcerated), the psychosocial barriers to HIV testing, including the possibility of being included in an additional stigmatized social category, may therefore be serious. Yet the consequences of late HIV diagnosis are grave, for individuals, their sexual partners, and their communities. Thus, to reduce the effect of drug use as a barrier to HIV testing for female HHR, multiple, simultaneous strategies are needed, some of which are already in place and others of which exist and could theoretically be implemented on a larger scale. These strategies include the normalization and routinization of HIV testing in health-care settings that serve women; providing testing in trusted community-based venues such as faith-based settings; anonymous HIV testing; home-based self-testing; policies and programs to reduce drug, HIV, and related stigmas; and providing harm reduction-based services to assist women to better manage substance use in order to reduce impediments to HIV testing (58, 59). The present study did not find recent drug use was a barrier to HIV testing for men, but their engagement in substance use treatment was associated with past-year HIV testing. There is good evidence that drug use affects men and women differently (60), and more research is needed to identify the gender-specific pathways from drug use to drug treatment to HIV testing and how the adverse effects of drug use can be ameliorated for both men and women.

### Individual/Attitudinal Barriers to HIV Testing

Women who ran out of money for basic necessities in the past year were more likely to have been tested for HIV, an unexpected finding. We speculate that those with serious financial hardship may have greater need for social services, which in turn leads them to settings where HIV testing is offered. Alternatively, serious deprivation may trigger survival-related behaviors and increased perceived risk of HIV, thereby motivating HIV testing (18). In a sensitivity analysis that excluded lifetime STI testing, general knowledge of HIV was associated with past-year testing in women but not men. A recent systematic review of psychological factors related to HIV testing similarly found a positive association, albeit small, between HIV knowledge and HIV testing (18), although it did not examine gender differences. Thus, while the present study highlights the importance of addressable, individual-level factors in HIV testing behavior such as HIV knowledge, taken together with the literature, it also underscores the complexity of health behavior, particularly among populations at-risk, and the need to examine the pathways to HIV testing separately for men and women.

### Social Factors and HIV Testing

Peer norms regarding HIV testing, that is the extent to which participants perceived their peers as supporting HIV testing for themselves, and with respect to the participant, were associated with HIV testing for males and females. This highlights the critical role perceptions of peers’ attitudes and actions have on one’s own behavior (25). Past studies have found unfavorable social norms about HIV are closely aligned to perceived HIV stigma (61). Indeed, although not directly examined in the present study, stigma continues to be a potent barrier to HIV testing and treatment, as detailed by Mahajan and colleagues in a recent review (62) and discussed above. Yet these authors note that our understanding of the role of stigma in HIV/AIDS is hampered by the absence of an explicit conceptualization, a comprehensive framework that includes a structural understanding of stigma and valid measures (62). Our research on HHR that takes structural and related emotional/attitudinal factors, such as fear and mistrust, into account is well suited to advance the literature on stigma, which will be explored in future studies.

### Structural Factors and Access to HIV Testing

Sexually transmitted infection testing can be considered a background factor, but such testing is embedded in health-care systems, and therefore, we consider the relationships among STI and HIV testing and access to medical settings together in this section. For both males and females, receiving testing for STIs over the lifetime, whether or not a diagnosis was confirmed, was associated with past-year HIV testing, as was their having been diagnosed with an STI in the past. We hypothesize that individuals concerned about STIs, for example, after engaging in behavior they considered risky, or experiencing symptoms of infection, present for STI testing in a setting where HIV testing is offered as well (63). Thus, this moment of heightened risk or perceived risk may motivate the acceptance of HIV testing, and also create the conditions for future HIV testing (64). Moreover, STIs, perhaps with the exception of HSV-2, tend to be less stigmatizing than HIV, which may encourage HHR to seek out STI testing in these types of circumstances (65), highlighting the important role of STI testing in achieving the goal of HIV elimination (66). Yet at present, the STI and HIV prevention systems in the US are considered “siloed” or largely separate, which may lead to missed opportunities for comprehensive sexual health services (67). In fact, public health system leaders in the US have called for better integration of STI and HIV services to improve the early diagnosis of a range of sexually transmitted health problems (63). Further, it is possible HIV testing triggered STI testing in some cases (68), and the cross-sectional nature of the data do not allow us to establish temporal order in this association.

Having tested in the past year was associated with involvement in medical and other systems (e.g., criminal justice and substance use treatment) where HIV testing is typically offered, but these associations varied by gender. The ability to access general medical care promoted recent HIV testing among females but not males, perhaps reflecting women’s regular engagement in gynecological and prenatal care, where HIV testing is normalized and routinely offered (1), and/or social norms regarding the need for regular gynecological health care among women (69). Among men, engagement in substance use treatment was associated with HIV testing. Indeed, these treatment settings may offer HIV testing on-site (70) and/or foster motivation for HIV testing (71). Further, receiving substance use treatment may comprise a type of structural intervention reducing other “competing priorities” while enhancing access to HIV testing. Men who had been incarcerated in their lives, but not in the past year, were less likely to have received past-year HIV testing, highlighting the ways in which incarceration both impedes HIV testing, for example, when past incarceration serves as a barrier to HIV testing access (72) or boosts HIV testing rates (for example, when men are tested in that setting) (73). On the other hand, most HHR will not encounter substance use treatment and criminal justice systems as regularly as health-care settings. Therefore, these systems alone are not sufficient to achieve sufficient provision of regular, annual HIV testing for HHR.

### Limitations

One limitation was the study’s cross-sectional design, which restricts causal inference. Further, additional unmeasured variables may be associated with HIV testing in this population, such as stigma, which was not assessed in this study but which is an important factor, as described above. The data are based on the quality of the RDS sampling approach described above, which may have missed key subpopulations of HHR that were not recruited by peers or who had a small probability of being recruited. This includes persons who decline to participate in HIV testing-related research studies because of distrust of research, fear of HIV testing, or HIV stigma. Finally, there may have been issues related to recall and social desirability biases in the reporting of HIV testing.

### Implications

Heterosexuals at high risk experience serious and multifaceted barriers to regular, annual HIV testing that vary to some extent by gender. The present study suggests a number of approaches that, taken together, have potential to address this gap. These include increasing the number and types of systems both male and female HHR encounter that offer high-quality HIV testing, with an emphasis on providing easy access to HIV testing, and developing culturally appropriate strategies to enhance readiness and motivation to test for HIV; insuring that CDC guidelines regarding annual HIV testing are widely known among HHR; promoting STI as well as HIV testing and better integrating these two largely separate systems; delivering active outreach, support, and tailored services for women who use drugs; and increasing general HIV knowledge to foster acceptance of HIV testing, particularly for women. Further, the present study highlights the potential utility of social/behavioral intervention approaches such as peer-driven intervention, which can be used to harness the positive influence of social networks in order to reduce unfavorable shared peer norms regarding HIV testing and thereby increase motivation for HIV testing (74, 75). Further, future research on policies and interventions to reduce stigma is recommended, as stigma is a critical impediment to regular, annual HIV testing among HHR (18), but effective strategies to reduce stigma have received insufficient attention in the literature to date.

## Author Contributions

MG conceived of the overall study concept and design and played a primary role in writing the manuscript. CC participated in the design of the study, planned the statistical analyses, and helped draft the manuscript. AK designed study procedures and helped draft the manuscript. NL participated in the design of the study and study implementation procedures. AR participated in the design of assessment and regulatory procedures. LL, MO, BM, AB, and TM-G participated in the design of recruitment, enrollment, and assessment procedures. All authors read and approved the final manuscript.

## Conflict of Interest Statement

The authors declare that the research was conducted in the absence of any commercial or financial relationships that could be construed as a potential conflict of interest.
